# Case Report: Response to Immunotherapy and Anti-Androgen Therapy in Male Occult Triple-Negative Breast Cancer

**DOI:** 10.3389/fonc.2022.840453

**Published:** 2022-03-30

**Authors:** Xin-Hua Wang, Jing Zhang, Jie Wu, Xiao-Han He, Yan-Ru Shen, Yong-Gang Peng, Yu-Zhi An

**Affiliations:** ^1^ Department of Oncology, The First Affiliated Hospital of Jinzhou Medical University, Jinzhou, China; ^2^ Department of Medical Science, Berry Oncology Corporation, Beijing, China

**Keywords:** male breast cancer, occult breast cancer, immunothearpy, antiandrogen therapy, case report

## Abstract

Male occult triple-negative breast cancer (TNBC) is an exceedingly rare form of breast cancer, and prospective information regarding its management is therefore lacking. Current treatment strategies are largely extrapolated from clinical trials of female breast cancer, leading to substantial knowledge gaps concerning the optimal management of male breast cancer. Here, we present a male patient with occult TNBC who responded to immunotherapy, with an obvious reduction in his tumor burden following antiandrogen therapy, after heavy treatment with several lines of chemotherapy. This case highlights the potential efficacy of immunotherapy in cases of male TNBC and suggests a role for antiandrogen therapy in managing patients with luminal androgen receptor-positive TNBC.

## Introduction

Occult breast cancer (OBC) is a specific type of breast cancer that usually manifests with axillary lymph node metastasis but no identifiable primary breast tumor. OBC accounts for approximately 1% of all breast cancers and male patients only comprise around 1% of cases of OBC (1). Given its rarity, there have been no prospective or retrospective studies concerning the management of male OBC, and the only published data comes from case reports. The only clinical trial (International Male Breast Cancer Program) of breast cancer focusing on men is still ongoing (2). The management of male patients is thus currently usually extrapolated from clinical trials enrolling female breast cancer patients, including treatments such as endocrine therapy, chemotherapy, and targeted therapy (3).

Most male breast cancers are hormone receptor-positive. Around 1% to 3.6% of male breast cancer patients have triple-negative breast cancer (TNBC) (2, 4), which is defined by the lack of expression of estrogen receptor, progesterone receptor, and human epidermal growth factor receptor 2. The systematic treatment for TNBC is mainly confined to chemotherapy currently, even though immunotherapy has been shown to improve OS in female patients with TNBC. The limited treatment options mean that the clinical outcomes of TNBC are usually worse than those for other breast cancers. To explore more potential treatment options, molecular subtyping based on gene expression profiles has been carried out and several studies have revealed at least four TNBC subtypes: luminal androgen receptor (LAR), mesenchymal, basal-like immunosuppressed, and basal-like immune-activated subtypes (5, 6). New pathways have been identified in these subtypes, and novel therapies targeting these pathways, including androgen receptor (AR) inhibitors, cyclin-dependent kinase inhibitors, and mammalian target of rapamycin inhibitors, are being studied in preclinical models and clinical trials (5), with promising results. However, the efficacies of these treatments need to be further confirmed, including their efficacies in male patients.

Given the limited availability of prospective data, joint efforts are needed to improve our understanding of the optimal management strategies for male TNBC, especially regarding immunotherapies or other emerging therapies. Here, we present the case of a male patient with occult TNBC and strong AR expression. The patient was treated with several lines of chemotherapy with limited efficacy, but subsequently achieved a favorable response to immunotherapy, followed by an obvious reduction in his tumor burden as a result of antiandrogen therapy including bicalutamide and goserelin.

## Case Presentation

In June 2019, a 64-year-old man discovered an enlarging, painful right axillary mass. His palpable lymph nodes were increased. He attended our hospital for treatment in July 2020. He denied any medical history of hypertension, diabetes, coronary heart disease, or infectious diseases, such as hepatitis and tuberculosis, and any family history of metabolic or genetic diseases.

**Figure 1:**
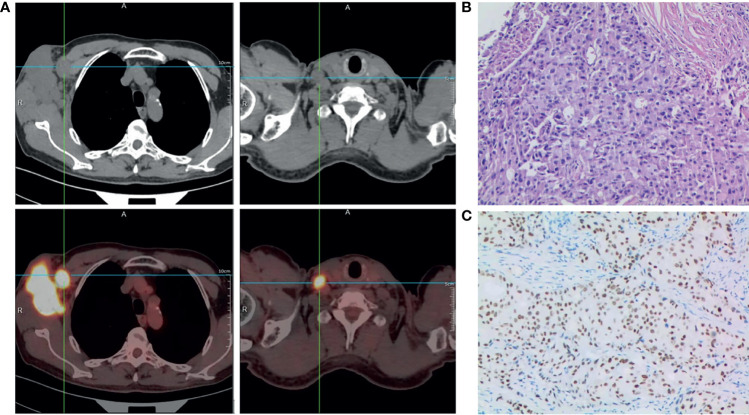
Diagnosis of male occult TNBC. **(A)** Positron emission tomography-computed tomography scan of the entire body showing abnormal metabolism in the right axilla and supraclavicular lymph node at diagnosis. **(B)** Representative histopathological image (hematoxylin and eosin staining) of needle biopsy specimen of the lesion in the right axilla; ×200. **(C)** Immunohistochemistry staining showing positive expression of androgen receptor (AR) in carcinoma cell nuclei. The AR-positivity index was 80%; ×200.

Physical examination in July 2020 revealed a hard mass measuring about 5.0×6.0×5.0 cm in his right axilla, with purple nodules but no surface ulceration. In addition, a palpable lymph node was detected on the right clavicle, measuring approximately 1.5×2.0 cm. His bilateral breasts were asymmetrical. There was no palpable breast mass and his nipples were not concave. Whole-body positron emission tomography-computed tomography (PET-CT) examination showed abnormal metabolism in the lymph nodes of his right axilla (**Figure 1A**) and in his supraclavicular lymph node, indicating metastasis (**Figure 1A**), but no abnormal metabolism in either breast or any other part of the body.

Histopathological examination of needle biopsy specimens of the right axilla mass revealed adenocarcinoma (**Figure 1B**). Immunohistochemical staining was strongly positive for CK7, GATA-3, GCDFP-15, Ki67 (~50%), and AR (80%), and negative for mammaglobin, estrogen receptor, progesterone receptor (5%), TP53 (~5%), CK5/6(−) and Her-2(0-1+), suggesting breast adenocarcinoma. Based on the above results, he was diagnosed with male occult TNBC (clinical stage IIIC [cTxN3M0]), after a multi-disciplinary panel discussion in our hospital. Subsequent molecular testing of the needle biopsy by a 654 gene panel sequencing revealed 28 mutations (**Supplementary Table 1**) (BerryOncology Inc.), but no mutations of cancer-predisposing germline genes, such as breast cancer susceptibility gene 1 *(BRCA1*) or breast cancer susceptibility gene 2 (*BRCA2*), were identified. The targeted gene sequencing (BerryOncology Inc.) revealed a high TMB with 19.43 mutations/Mb and microsatellite stable (MSS). Programmed death-ligand-1 (PD-L1) expression was evaluated by 22C3 PD-L1 immunohistochemical assay on the Dako Link-48 platform. It showed a combined positive score (CPS) of 5.

**Figure 2:**
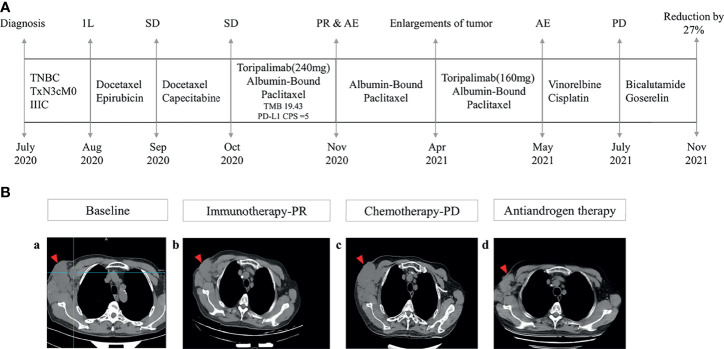
Management flow and treatment response evaluation. **(A)** Schematic of course of disease management. **(B)** Tumor response assessment and follow-up. Computed tomography images of right axilla tumor **(a)** at baseline (October 2020), **(b)** after immunotherapy (March 2021), **(c)** under chemotherapy (July 2021), and **(d)** under antiandrogen therapy consisting of bicalutamide and goserelin (November 2021) 1L, first line; SD, stable disease; PR, partial response; AE, adverse events; PD, progressive disease.

**Figure 3:**
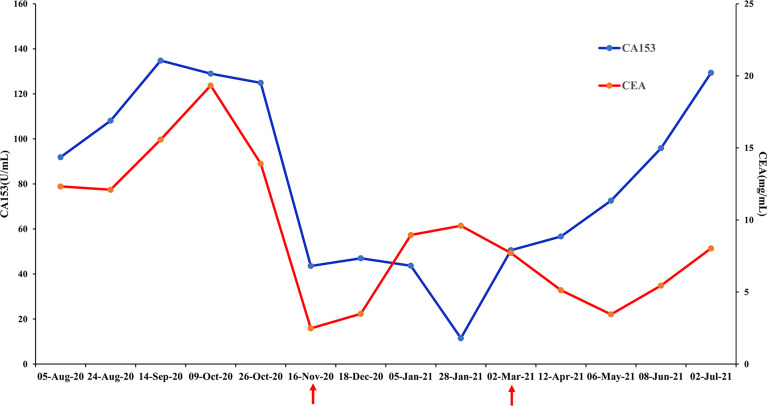
Changes in breast cancer tumor biomarkers, carcinoembryonic antigen (CEA) and CA153. Normal range: CEA<5 mg/mL, CA153<31.3 U/mL.

**Figure 4:**
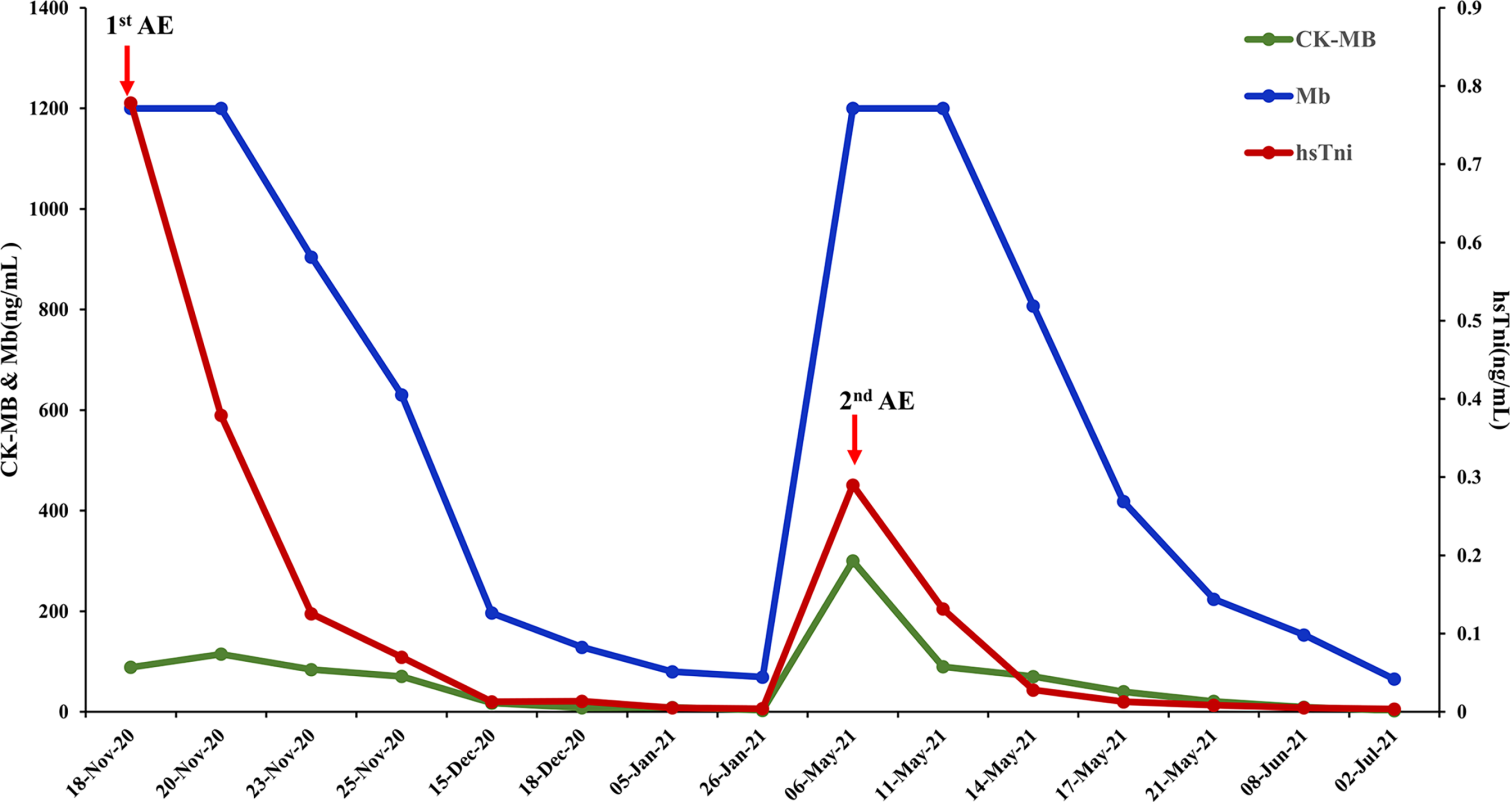
Biomarker changes in relation to immunotherapy-related adverse events. Normal range: CK-MB< 7.2 ng/mL, Mb<154.9 ng/mL, hsTni<0.0342ng/mL CK-MB, creatine kinase-MB; Mb, myoglobin; hsTni, high-sensitivity troponin I; AE, adverse events.

The patient received chemotherapy in August 2020 (**Figure 2A**), as follows: docetaxel 75 mg/m^2^ plus epirubicin 90 mg/m^2^ (day 1, 21 days per cycle). The right axillary mass failed to shrink after two cycles and his tumor biomarkers (carcinoembryonic antigen and CA153) continued to increase. The patient switched to docetaxel 75 mg/m^2^ plus capecitabine 1000 mg/m^2^ (day 1, 21 days per cycle), with no reduction in tumor burden. Several of the purple nodules fused and became ulcerated. In October 2020, the patient was switched to immunotherapy in combination with chemotherapy (toripalimab 240 mg plus albumin-bound paclitaxel 300 mg, day 1). An early and notable tumor response to toripalimab was observed within the first two cycles of immunotherapy; the ulcers healed and there was local redness and swelling on the surface but no fused nodules in the right axilla. CT examination revealed a partial response to immunotherapy according to RECIST version 1.1, with a 44% reduction in tumor burden from 7.7 to 4.4 cm (**Figures 2Ba, b**). This clinical response was accompanied by a rapid decline in tumor markers to within the normal ranges (**Figure 3**). During immunotherapy, the patient developed grade 4 immune-related adverse events including hepatitis and myocarditis, but his symptoms were dramatically resolved after treatment with 80 mg methylprednisolone intravenously for 4 days, subsequently reduced to 40 mg. His troponin, myoglobin, and alanine aminotransferase levels gradually returned to within the normal ranges (**Figure 4**) and his clinical symptoms disappeared.

The patient discontinued immunotherapy in November 2020, but continued to receive albumin-bound paclitaxel, and bevacizumab 300 mg was added to the chemotherapy in January 2021. However, an obvious enlargement of his tumor was observed. The patient requested an another round of immunotherapy to control his disease development. Immunotherapy was restarted in April 2021 and a low dose (160mg) was adopted to reduce the risk of severe adverse events for this patient. It was stopped again, due to his high intolerance to toripalimab. Chemotherapy including vinorelbine and cisplatin was started in May 2021, but CT revealed an increase in his tumor burden to 6.6 cm in July 2021 (**Figures 2Bc**), and his response to chemotherapy was evaluated as progressive disease.

Because of the tumor’s strong androgen receptor-positivity (80%) **Figure 1C**) and the patient’s progressive disease, we considered additional options. The patient was started on antiandrogen therapy consisting of bicalutamide 50 mg/day plus goserelin 3.6 mg every 4 weeks in July 2021. His tumor burden decreased from 6.6 cm to 4.8 cm following antiandrogen therapy (**Figures 2Bd**), and notable radiologic improvement was maintained without progression for 4 months. The treatment was well tolerated, with no reported side effects.

## Discussion

OBC in men is rare. Most case reports of occult metastatic breast cancer involve hormone receptor-positive cases, while the incidence of male TNBC is around 1–3.6% (2, 4). The rarity of the disease means that there have been almost no case reports to guide the management of male TNBC. To the best of our knowledge, the current, extremely rare case represents the first case report focusing on the clinical management of occult male TNBC, including both immunotherapy and antiandrogen therapy. Given the lack of prospective data on the management of male breast cancer, its current standard treatment is almost the same as that for female breast cancer. One different point is that genetic testing for cancer predisposing genes is recommended for all male patients by the NCCN guidelines. For the current patient, no germline mutations of cancer susceptibility genes were detected, including *BRAC1*, *BRAC2*, partner and localizer of BRCA2 (*PALB2*), checkpoint kinase 2 (*CHEK2*) and ataxia telangiectasia mutated (*ATM*).

This patient was diagnosed with stage IIIC breast cancer and his tumor is unresectable at diagnosis. Chemotherapy was given at first to decrease his tumor burden and control his disease development. TNBC is usually chemosensitive; however, the current patient showed no reduction in tumor burden following treatment with either docetaxel plus epirubicin or docetaxel plus capecitabine. The IMpassion130 trial has shown that PD-L1-positive advanced TNBC patients can gain clinical benefits from the combination therapy of atezolizumab and albumin-bound paclitaxel (7). In addition, pembrolizumab has been approved for treating patients with unresectable or metastatic TMB-H (≥10 mutations/Mb) or microsatellite instability-high solid tumors. Given the limited clinical benefits of chemotherapy, the promising efficacy of immunotherapy and high TMB (19.43 mutations/Mb), the patient was switched to immunotherapy. Toripalimab, which is an antibody for PD-1, was used to treat this patient, considering the cost-effectiveness. A good tumor response was observed. The high TMB value of his tumor may explain the responses to immunotherapy, despite MSS and a negative PD-L1 test result (CPS, cut-off ≥10) as concluded from the NCCN Guidelines (Version 1, 2022) for breast cancer. Although immunotherapy was approved for the treatment of advanced or metastatic TNBC, no studies or case reports have examined its efficacy in cases of male TNBC. To the best of our knowledge, this study provides the first report demonstrating the efficacy of immunotherapy in male TNBC and necessity of gene test for male breast cancer treatment.

Given the high heterogeneity of TNBC, further molecular subtyping is necessary to allow its optimal management. Multiple distinct subtypes of TNBC have been identified, among which LAR TNBC has shown consistent findings in several studies, accounting for 13%–37% of cases of TNBC (8). This subtype is enriched in AR expression and androgen signaling pathways (9). The current patient’s tumor showed high AR expression (approximately 80%), and was therefore identified as LAR TNBC. LAR TNBC is characterized by a higher mutational burden compared with other types of TNBC (5, 10). This was consistent with the high TMB detected by a target sequencing panel in our patient. LAR TNBC is also characteristically inherently resistant to chemotherapy (11, 12), which was also observed in the present case, with no reduction in tumor burden under any chemotherapy regime, and the development of progressive disease after heavy chemotherapy in July 2021. This inherent resistance to chemotherapy may be one reason why patients with LAR TNBC have poorer clinical outcomes than other TNBC patients (10).

Antiandrogen therapies have been developed targeting the androgen signaling pathway in LAR TNBC, with some promising results. The nonsteroidal antiandrogen bicalutamide has been evaluated in clinical trials in breast cancer patients (13). It competitively inhibits the binding of androgens to the AR, and has also been successfully applied for the treatment of locally advanced and metastatic prostate cancer, either as monotherapy or combined with a gonadotropin-releasing hormone agonist (14, 15). The current patients received AR inhibition combined with luteinizing hormone-releasing hormone agonists (bicalutamide plus goserelin), leading to an obvious and durable reduction of tumor burden. His progressive disease under chemotherapy was effectively controlled by antiandrogen therapy, with continued response to date. In contrast, another patient with occult male TNBC died due to the failure of chemotherapy to control his disease (16). Immunohistochemistry showed that this patient’s tumor was also positive for AR expression, but the test was carried out retrospectively, and the patient was therefore unable to benefit from antiandrogen therapy. These cases highlight the importance of molecular subtyping of TNBC to ensure its optimal management, and suggest a role for antiandrogen therapy in the management of LAR TNBC. In addition, the possible adverse effects of anti-androgen therapy in men should be considered and interventions should be taken if necessary during the treatment. The potential adverse effects include hot flushes, cardiovascular disease, decrease of libido, impairment of sexual and cognitive functions, unfavourable metabolic changes, dementia, loss of bone density and bone fracture, fatigue and so on (17).

It is important to note that a phase II clinical trial of AR inhibitors showed limited clinical benefit in female TNBC patients (18), possibly due to the low cut-off for AR expression of about 10%. Notably, the AR expression rate in the current patient was >80% **Figure 1C**), suggesting high dependence on AR signaling pathways for tumor survival and proliferation. One previous case report suggested a durable response of AR-positive male TNBC to goserelin (19), while another suggested a complete response of metastatic LAR TNBC to bicalutamide in a female patient (20). Notably, the AR expression rates in both these patients were 100%. In addition, a recent phase II clinical trial found that a high AR expression rate was correlated with a higher response rate to AR inhibitors in patients with TNBC (21). The clinical benefit rate for tumors with AR expression >40% was 80%, compared with 18% for tumors with an expression rate < 40% (p< 0.0001). These results, together with the present case, indicate the need for a standardized cut-off for the detection of AR expression in order to stratify TNBC patients likely to benefit from AR inhibitor therapy.

## Conclusion

We present a case of male occult AR-positive TNBC who showed good responses to immunotherapy and obvious reduction of his tumor burden after antiandrogen therapy, after heavy treatment with several lines of chemotherapies. To the best of our knowledge, this case provides the first report of the efficacy of immunotherapy in male occult TNBC. In addition, the effective control of disease progression achieved by antiandrogen therapy with minimal toxicity indicates the need for molecular subtyping of TNBC, and the role of antiandrogen therapy in managing LAR TNBC. Given the treatment response in this case, we suggest that further research involving similar cases is needed to confirm the benefits of immunotherapy and antiandrogen therapy in patients with male TNBC.

## Author’s Note

The manuscript was prepared and revised according to the CARE Checklist (2016).

## Data Availability Statement

The raw data supporting the conclusions of this article will be made available by the authors, without undue reservation.

## Ethics Statement

Written informed consent was obtained from the individual(s) for the publication of any potentially identifiable images or data included in this article.

## Author Contributions

X-HW and Y-ZA contributed to patient management and histological evaluation. JW and Y-ZA contributed to technical and material support. Y-ZA designed and reviewed the report. X-HW wrote the manuscript. X-HH, Y-RS, and Y-GP reviewed and corrected the manuscript. X-HH, Y-RS, and Y-GP analyzed the data. All authors contributed to revision for important intellectual content and approved the final version of the manuscript.

## Conflict of Interest

Authors X-HH, Y-RS, and Y-GP were employed by Berry Oncology Corporation.

The remaining authors declare that the research was conducted in the absence of any commercial or financial relationships that could be construed as a potential conflict of interest.

## Publisher’s Note

All claims expressed in this article are solely those of the authors and do not necessarily represent those of their affiliated organizations, or those of the publisher, the editors and the reviewers. Any product that may be evaluated in this article, or claim that may be made by its manufacturer, is not guaranteed or endorsed by the publisher.

## References

[1] Siegel RL Miller KD Jemal A. Cancer Statistics 2020. CA Cancer J Clin (2020) 70:7–30. doi: 10.3322/caac.21590 31912902

[2] Cardoso F Bartlett JMS Slaets L Van Deurzen CHM Van Leeuwen-Stok E Porter P. Characterization of Male Breast Cancer: Results of the EORTC 10085/TBCRC/BIG/NABCG International Male Breast Cancer Program. Ann Oncol (2018) 29:405–17. doi: 10.1093/annonc/mdx651 PMC5834077 29092024

[3] Hassett MJ Somerfield MR Baker ER Cardoso F Kansal KJ Kwait DC. Management of Male Breast Cancer: ASCO Guideline. J Clin Oncol (2020) 38:1849–63. doi: 10.1200/JCO.19.03120 32058842

[4] Yadav S Karam D Bin Riaz I Xie H Durani U Duma N. Male Breast Cancer in the United States: Treatment Patterns and Prognostic Factors in the 21st Century. Cancer (2020) 126:26–36. doi: 10.1002/cncr.32472 31588557 PMC7668385

[5] Marra A Trapani D Viale G Criscitiello C Curigliano G. Practical Classification of Triple-Negative Breast Cancer: Intratumoral Heterogeneity, Mechanisms of Drug Resistance, and Novel Therapies. NPJ Breast Cancer (2020) 6:54. doi: 10.1038/s41523-020-00197-2 33088912 PMC7568552

[6] Jiang Y-Z Liu Y Xiao Y Hu X Jiang L Zuo W-J. Molecular Subtyping and Genomic Profiling Expand Precision Medicine in Refractory Metastatic Triple-Negative Breast Cancer: The FUTURE Trial. Cell Res (2021) 31:178–86. doi: 10.1038/s41422-020-0375-9 PMC8027015 32719455

[7] Schmid P Adams S Rugo HS Schneeweiss A Barrios CH Iwata H. Atezolizumab and Nab-Paclitaxel in Advanced Triple-Negative Breast Cancer. N Engl J Med (2018) 379:2108–21. doi: 10.1056/NEJMoa1809615 30345906

[8] Collins LC Cole KS Marotti JD Hu R Schnitt SJ Tamimi RM. Androgen Receptor Expression in Breast Cancer in Relation to Molecular Phenotype: Results From the Nurses' Health Study. Modern Pathol (2011) 24:924–31. doi: 10.1038/modpathol.2011.54 PMC3128675 21552212

[9] Lehmann BD Bauer JA Chen X Sanders ME Chakravarthy AB Shyr Y. Identification of Human Triple-Negative Breast Cancer Subtypes and Preclinical Models for Selection of Targeted Therapies. J Clin Invest (2011) 121:2750–67. doi: 10.1172/JCI45014 PMC3127435 21633166

[10] Bareche Y Venet D Ignatiadis M Aftimos P Piccart M Rothe F. Unravelling Triple-Negative Breast Cancer Molecular Heterogeneity Using an Integrative Multiomic Analysis. Ann Oncol (2018) 29:895–902. doi: 10.1093/annonc/mdy024 29365031 PMC5913636

[11] Lehmann BD Jovanović B Chen X Estrada MV Johnson KN Shyr Y. Refinement of Triple-Negative Breast Cancer Molecular Subtypes: Implications for Neoadjuvant Chemotherapy Selection. PloS One (2016) 11:e0157368. doi: 10.1371/journal.pone.0157368 27310713 PMC4911051

[12] Masuda H Baggerly KA Wang Y Zhang Y Gonzalez-Angulo AM Meric-Bernstam F. Differential Response to Neoadjuvant Chemotherapy Among 7 Triple-Negative Breast Cancer Molecular Subtypes. Clin Cancer Res (2013) 19:5533–40. doi: 10.1158/1078-0432.CCR-13-0799 PMC3813597 23948975

[13] Moelans CB De Ligt J Van Der Groep P Prins P Besselink NJM Hoogstraat ND. The Molecular Genetic Make-Up of Male Breast Cancer. Endocr Relat Cancer (2019) 26:779–94. doi: 10.1530/ERC-19-0278 PMC6938562 31340200

[14] Pilepich MV Winter K John MJ Mesic JB Sause W Rubin P. Phase III Radiation Therapy Oncology Group (RTOG) Trial 86-10 of Androgen Deprivation Adjuvant to Definitive Radiotherapy in Locally Advanced Carcinoma of the Prostate. Int J Radiat Oncol Biol Phys (2001) 50:1243–52. doi: 10.1016/S0360-3016(01)01579-6 11483335

[15] Wirth MP Hakenberg OW Froehner M. Antiandrogens in the Treatment of Prostate Cancer. Eur Urol (2007) 51:306–14. doi: 10.1016/j.eururo.2006.08.043 17007995

[16] Angelats L Estival A Martinez-Cardús A Musulen E Margelí M. Occult Triple Negative Male Breast Cancer. The Usefulness of Molecular Platforms. A Case Report. Curr Problems Cancer: Case Rep (2021) 4:100097. doi: 10.1016/j.cpccr.2021.100097

[17] Nguyen PL Alibhai SM Basaria S D'amico AV Kantoff PW Keating NL. Adverse Effects of Androgen Deprivation Therapy and Strategies to Mitigate Them. Eur Urol (2015) 67:825–36. doi: 10.1016/j.eururo.2014.07.010 25097095

[18] Gucalp A Tolaney S Isakoff SJ Ingle JN Liu MC Carey LA. Phase II Trial of Bicalutamide in Patients With Androgen Receptor–Positive, Estrogen Receptor–Negative Metastatic Breast Cancer. Clin Cancer Res (2013) 19:5505–12. doi: 10.1158/1078-0432.CCR-12-3327 PMC4086643 23965901

[19] Abdel Azim H Kassem L Shohdy KS Eshaak B Anis SE Kamal NS. Durable Response of Androgen Receptor-Positive Male Breast Cancer to Goserelin. J Breast Cancer (2019) 22:141–8. doi: 10.4048/jbc.2019.22.e2 PMC6438837 30941241

[20] Arce-Salinas C Riesco-Martinez MC Hanna W Bedard P Warner E. Complete Response of Metastatic Androgen Receptor-Positive Breast Cancer to Bicalutamide: Case Report and Review of the Literature. J Clin oncology: Off J Am Soc Clin Oncol (2014) 34:e21–4. doi: 10.1200/JCO.2013.49.8899 24888812

[21] Palmieri C Linden HM Birrell S Lim E Schwartzberg LS Rugo HS. Efficacy of Enobosarm, a Selective Androgen Receptor (AR) Targeting Agent, Correlates With the Degree of AR Positivity in Advanced AR+/estrogen Receptor (ER)+ Breast Cancer in an International Phase 2 Clinical Study. J Clin Oncol (2021) 39:1020. doi: 10.1200/JCO.2021.39.15_suppl.1020

