# Supplementary material for: Case Report: Response to Immunotherapy and Anti-Androgen Therapy in Male Occult Triple-Negative Breast Cancer
Source: Front Oncol. 2022 Mar 30;12:840453. doi: 10.3389/fonc.2022.840453 (PMC9005963; doi:10.3389/fonc.2022.840453)
Supplement: Supplementary file 1 [file Table_1.pdf]

Supplementary Table 1. Somatic mutations detected in the tumor sample

Gene	Chromosome	Exon	Amino Acid Change	Base Change	Ratio	Type	Transcript	Annotation
ERBB2	chr17	21	p.L841V	c.2521C>G	0.3092	SNV	NM_004448.3	nonsynonymous SNV
TP53	chr17	9	p.Q331*	c.991C>T	0.1553	SNV	NM_000546.5	stopgain
TP53	chr17	4	p.D42*	c.123dupT	0.1686	Indel	NM_000546.5	stopgain
APC	chr5	16	p.D2430H	c.7288G>C	0.2086	SNV	NM_000038.5	nonsynonymous SNV
ARID1A	chr1	14	p.D1193H	c.3577G>C	0.1999	SNV	NM_006015.4	nonsynonymous SNV
BCORL1	chrX	3	p.G182A	c.545G>C	0.2159	SNV	NM_021946.4	nonsynonymous SNV
BTG1	chr12	2	p.Q135*	c.403C>T	0.1455	SNV	NM_001731.2	stopgain
EPHB1	chr3	5	p.E341Q	c.1021G>C	0.2596	SNV	NM_004441.4	nonsynonymous SNV
ERBB2	chr17	18	p.E698V	c.2093A>T	0.3057	SNV	NM_004448.3	nonsynonymous SNV
FANCM	chr14	14	p.E1138Q	c.3412G>C	0.0321	SNV	NM_020937.3	nonsynonymous SNV
FANCM	chr14	23	p.R2024I	c.6071G>T	0.0966	SNV	NM_020937.3	nonsynonymous SNV
FLT4	chr5	18	p.D874N	c.2620G>A	0.1684	SNV	NM_182925.4	nonsynonymous SNV
GRM3	chr7	4	p.A575D	c.1724C>A	0.0335	SNV	NM_000840.2	nonsynonymous SNV
KDM5A	chr12	5	p.L197F	c.589C>T	0.1397	SNV	NM_001042603.2	nonsynonymous SNV
KMT2B	chr19	33	p.Q2483*	c.7447C>T	0.1417	SNV	NM_014727.2	stopgain
MAX	chr14	3	p.S75L	c.224C>T	0.0957	SNV	NM_001271068.1	nonsynonymous SNV
MUC16	chr19	3	p.E8848Q	c.26542G>C	0.2223	SNV	NM_024690.2	nonsynonymous SNV
NFE2L2	chr2	5	p.F328L	c.984C>G	0.1627	SNV	NM_006164.4	nonsynonymous SNV
NFE2L3	chr7	4	p.S431F	c.1292C>T	0.1436	SNV	NM_004289.6	nonsynonymous SNV
POLQ	chr3	16	p.R1064T	c.3191G>C	0.1406	SNV	NM_199420.3	nonsynonymous SNV
RAD54L	chr1	5	splicing	c.271+2T>C	0.146	SNV	NM_001142548.1	splicing
RECQL4	chr8	11	p.G588V	c.1763G>T	0.0787	SNV	NM_004260.3	nonsynonymous SNV
RECQL4	chr8	10	p.E563*	c.1687G>T	0.0784	SNV	NM_004260.3	stopgain
RELN	chr7	13	p.D493N	c.1477G>A	0.1885	SNV	NM_005045.3	nonsynonymous SNV
SPEN	chr1	11	p.S760Qfs*49	c.2277delC	0.187	Indel	NM_015001.2	frameshift deletion
TYK2	chr19	7	p.E273Q	c.817G>C	0.0391	SNV	NM_003331.4	nonsynonymous SNV
ZFHX3	chr16	2	p.E59K	c.175G>A	0.0627	SNV	NM_006885.3	nonsynonymous SNV
ERBB4->IKZF2	chr2:213403173;chr2:213872808				0.850649	Fusion		
